# A Decrease in Lactobacilli in the Vaginal Microbiota Is Independently Associated With HPV Persistence in Women With High-Risk HPV Infection

**DOI:** 10.7759/cureus.50907

**Published:** 2023-12-21

**Authors:** Elif Avsaroglu, Babur Kaleli, Derya Kilic, Ilknur Kaleli, Tolga Guler

**Affiliations:** 1 Obstetrics and Gynecology, Denizli State Hospital, Denizli, TUR; 2 Obstetrics and Gynecology, Pamukkale University, Denizli, TUR; 3 Microbiology, Pamukkale University, Denizli, TUR

**Keywords:** hsil, persistence, lactobacilli, human papillomavirus, cervicovaginal microbiota

## Abstract

Background

Vaginal dysbiosis, an imbalance between species, can initiate some local changes in immune and metabolic signaling causing chronic inflammation. The mechanism of the clearance or progression of the HPV infection has not been uncovered yet. We hypothesized that vaginal dysbiosis may contribute to the persistence of the cervical HPV infection. Therefore we aimed to determine the association of lactobacillus dominancy index with cervical HR-HPV persistence.

Methods

A total of 100 women who were followed up because of high-risk HPV infection were defined as the target study group. The patients were evaluated in two groups; HPV positive (group with HPV persistence, n=43) and HPV negative (group with HPV clearance, n=57). Cervicovaginal swab samples and blood samples were evaluated for Nugent score, lactobacillus dominance, and white blood cell count. Statistical tests were performed by the IBM Statistical Product and Service Solutions (version 22, IBM SPSS Statistics for Windows, Armonk, NY) program. The continuous variables were presented using the mean±standard deviation (SD), and the categorical variables were presented as the number of cases and their percentage. A p value less than 0.05 (<0.05) was set as statistically significant.

Results

HPV persistence was observed in 43 (43%) patients. Univariate analysis revealed that age, menopausal status, and lactobacillus reduction were associated with HPV persistence (p<0.05). The median value of the Nugent score was similar among groups. After logistic regression analysis, lactobacillus reduction continued to be associated with HPV persistence, independent of age and menopause (OR: 2.668, 96% CI: 1.069-6.662, p<0.05)

Conclusions

A decrease in lactobacilli in the cervicovaginal microbiota is associated with the persistence of HPV, regardless of age and menopausal status in this study.

## Introduction

The vaginal microbiota, including various colonies in this specific environment, plays an important role in reproductive health [1]. As with other mucous membranes in the human body, the female lower reproductive system has its own microbiota, and a healthy cervicovaginal microbiome is dominated by one or more Lactobacillus species [2]. Vaginal dysbiosis, an imbalance between species, can initiate some local changes in immune and metabolic signaling causing chronic inflammation. These changes may result in some medical conditions such as bacterial vaginosis, pelvic inflammatory disease, miscarriage, and preterm birth [3].

Cervicovaginal microbiome changes in patients with cervical precancerous lesions are currently under investigation. As is known, human papillomavirus (HPV) infection is essential for the development of cervical cancer, with a lifetime prevalence of 70% [4], and persistent high-risk HPV infection (HR-HPV) is the main risk factor for progression to carcinogenesis [5]. Although HR-HPV has evolved several mechanisms to evade the host's immune system such as secreting lower amounts of biochemicals and guiding the antigen-processing cells [6], clearance occurs in most infections within two years that does not impose a further cancer risk [7].

Certain factors, such as younger age, premenopausal status, no smoking, and monogamy are associated with a significantly higher proportion of HPV clearance [7]. However, the underlying mechanism of the regression or progression of the HPV infection has not been uncovered yet. More recently, it has been revealed that vaginal dysbiosis may contribute to carcinogenesis by potentiating the effect of HPV infection [8]. Improved knowledge in this field could clarify how the microbiome is effective in the progression or regression of persistent HPV infection and which features of the microbiome are expected to develop precancerous lesions. The present study aimed to investigate the association of the lactobacillus dominancy index and bacterial colonization with cervical HR-HPV persistence. This novel information might lead us to avoid unnecessary invasive examinations and to develop interventions such as probiotics that may provide us with a better approach to preserve the cervix during the management of cervical HPV infections.

## Materials and methods

Study design

The study population comprised women who had previously undergone colposcopic examination because of a documented high-risk human papillomavirus (HR-HPV) infection. The patients were between the ages of 30 and 65. Those with complete follow-up information available after colposcopy were included in the study. These cases were invited to participate in this study while attending their routine follow-up visit. Before initiating the study, ethical committee approval was obtained from the University Hospital Clinical Research Ethics Committee (11/08/2020- no:15). All patients were informed in detail about the study, and their written informed consent was obtained.

At the first year follow-up, the patients were evaluated in two groups; HPV positive (group with HPV persistence) and HPV negative (group with HPV clearance). Cervicovaginal swab samples were taken from individuals who agreed to participate in the study during routine gynecological examination. These samples were delivered to the microbiology laboratory immediately after collection.

Demographic data, anamnesis, and previous medical records of the patients were obtained through the gynecological patient evaluation form and file records. The HPV status of the patients before the colposcopy and at the first-year follow-up visit was noted. The Abbott Real-Time High-Risk HPV (HR-HPV) test was used to detect HR-HPV in HPV tests. As a result of this test, HPV-16, HPV-18, and other high-risk groups (OHR) are reported as positive results. Other follow-up information and laboratory results were accessed through file records.

Exclusion criteria

Exclusion criteria from the study were determined as follows: i) active vaginal infection; ii) vaginal bleeding of unknown etiology; iii) history of other types of cancer; iv) known immunosuppressive disease or drug use; and vi) patients with high-grade lesions during final colposcopic evaluation.

Collection of samples

Cervicovaginal swab samples were collected in the outpatient clinic. This was done with patients in the lithotomy position, using a sterile vaginal speculum and a sterile 12x150 mm polypropylene swab. These swabs were inserted into a transportation tube containing a Stuart medium. Afterward, these samples were delivered to the microbiology laboratory immediately.

After the samples were delivered to the microbiology laboratory, the quality of the samples, Nugent score, and lactobacillus dominance were evaluated by two independent technicians who were blinded to the study group.

Nugent score assessment

Gram staining was performed on swab samples taken with “BTR sterile Stuart transport swab,” lactobacillus count from these samples and scored according to the Nugent scoring system defined [9]. The Nugent scoring system combines bacterial morphotypes together with Gram-staining properties to define the overall character of the vaginal microbiome [9].

The scoring system occurs briefly on a scale of 0 to 10 and has three components:

1) A score of 0-4 reflecting the presence of rod-shaped Gram-positive lactobacilli where 0 indicates the highest numbers; 2) A score of 0 to 4 reflecting the numbers of Gram-negative and Gram-variable bacteria where 4 indicates the highest numbers; and 3) a score of 0-2 reflecting the presence of curved rods.

Statistical method

Data were analyzed using the IBM Statistical Product and Service Solutions (version 22, IBM SPSS Statistics for Windows, Armonk, NY) program. Statistical values such as arithmetic mean, standard deviation, and median were analyzed for continuous variables; percentage and frequency were also analyzed for categorical variables. Evaluation of the numerical data was performed with the Student's T-test. The chi-square test was used for the relationship between categorical variables. Statistical significance was accepted as p<0.05. A multivariable logistic regression was performed to define the independent associations.

## Results

A total of 100 patients were included in the study. Demographic data and clinical features of all cases are shown in Table 1. The analysis of the cases was compared in two main groups at the first-year follow-up: the group without the persistence of HPV (n=57) and the group with the persistence of HPV (n=43). The demographic and clinical characteristics of the cases were also compared within these two groups and are shown in Table 2. In this comparison, both groups were similar in parity, age at marriage, number of partners, smoking, presence of diabetes, history of oral contraceptive use, and HPV type 16/18 positivity.

**Table 1:** Basic demographic and clinical features of the patients participating in the study OC: Oral contraceptives

Demographic and clinical features	Study group (N=100)
Age (average ± SD)	42.6 ± 9.3
Parity (average ± SD)	1.9 ± 1.8
Age at marriage (average ± SD)	20.5 ± 3.4
Number of partners >1 (n (%))	16 (16 %)
Smoking (n (%))	31 (31%)
Presence of diabetes (n (%))	13 (13%)
History of OC use (n (%))	19 (19%)
Menopause (n (%))	26 (26%)
History of HPV type 16/18 positivity (n (%))	67 (67%)

The mean age in the HPV-persistent group was significantly higher than in the nonpersistent group (45.0±10.7 vs. 40.7±7.7, p=0.03). Additionally, the proportion of women with menopause in the persistent group was found to be significantly higher than in the nonpersistent group (39.5% vs 15.8%, p=0.007).

The relationship between lactobacillus reduction and HPV persistence in the cervicovaginal swab sample was evaluated and shown in Table 3. HPV persistence was observed in 43 (43%) patients in total, and HPV persistence was found to be more common in the group with lactobacilli depletion. Although a decrease in the number of lactobacilli was observed in 21.1% of women in the group without the persistence of HPV, this rate was found to be significantly higher in the group with the persistence of HPV (46.5%, p=0.007).

**Table 2:** The basic demographic and clinical features of the patients included in the study, separately for groups with and without HPV persistence HPV: Human papillomavirus; OC; oral contraceptives *p<0.05

Demographic and clinical features	Without HPV persistence	With HPV persistence	P value
Age (average ± SD)	40.7 ± 7.7	45.0 ± 10.7	0.03*
Parity (average ± SD)	1.9 ± 1.0	1.8 ± 1.2	0.732
Age at marriage (average± SD)	20.7 ± 3.8	20.3 ± 2.9	0.604
Number of partners >1 (n (%))	6 (10.5%)	10 (23.3%)	0.086
Smoking (n (%))	16 (32%)	15 (40.5%)	0.411
Presence of diabetes (n (%))	7 (12.3%)	6 (14%)	0.805
History of OC use (n (%))	12 (21.1%)	7 (16.3%)	0.547
Menopause (n (%))	9 (15.8%)	17 (39.5%)	0.007*
History of HPV type 16/18 positivity (n (%))	37 (64.9%)	30 (69.8%)	0.609

**Figure 1:**
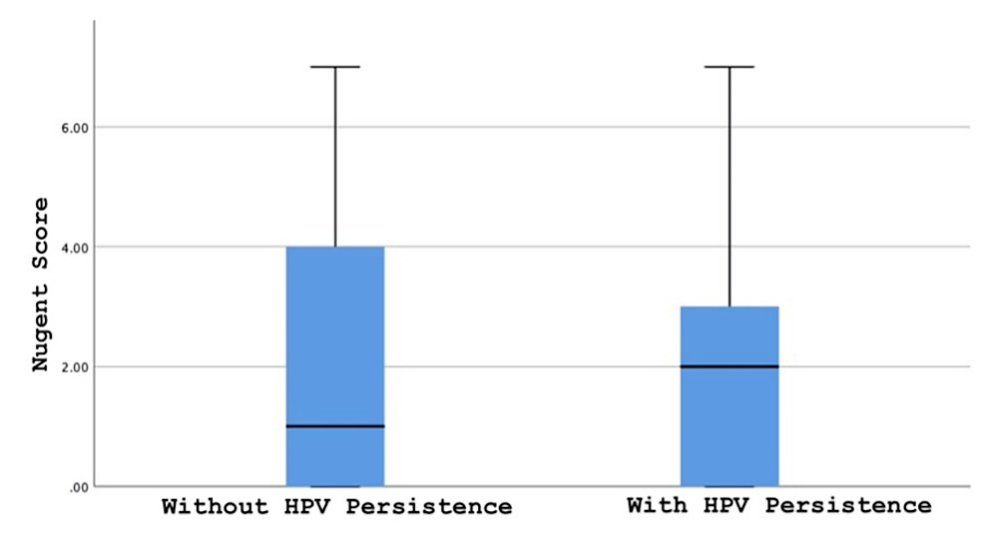
Comparison of Nugent score median value between two groups with and without HPV persistence

The Nugent score obtained from the cervicovaginal swab samples of the patients was compared in two groups with and without HPV persistence. When the median value was compared between the two groups in terms of the Nugent score (1 vs 2, p=0.745), no significant differences were observed (Figure 1).

**Figure 2:**
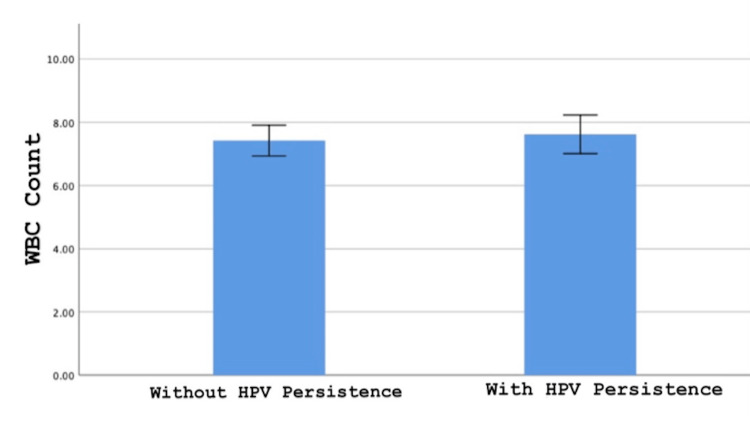
Comparison of white blood cell count median value between two groups with and without HPV persistence

Additionally, it was also evaluated whether there was a difference in white blood cell count between the two groups with and without HPV persistence. While the mean white blood cell count was 7.4±1.8 in the group without HPV persistence, it was 7.6±1.9 in the group with HPV persistence, which was statistically insignificant (p=0.610) (Figure 2).

Age, menopausal status, and lactobacillus reduction, which are the parameters that were found to be significant in terms of HPV persistence, were then analyzed by logistic regression analysis. As This analysis showed that lactobacillus reduction was significantly associated with HPV persistence, independent of age and menopause (Table 4).

**Table 3:** Relationship between lactobacillus reduction and persistence of HPV HPV: Human papillomavirus *p<0.05

	Without HPV persistence n=57 (100%)	With HPV persistence n=43 (100%)	P value
Normal Lactobacillus count (n=68)	45 (78.9%)	23 (53.5%)	0.007
Decreased Lactobacillus count (n=32)	12 (21.1%)	20 (46.5%)	

**Table 4:** Logistic regression analysis results for HPV persistence

Parameters	B	Standard error	Odds ratio	95% confidence interval for the odds ratio	P value
Constant	-1.412	1.525	0.244		0.354
Age	0.014	0.039	1.014	0.940-1.095	0.716
Menopause	0.774	0.833	2.169	0.424-11.105	0.353
Lactobacillus decrease	0.982	0.467	2.668	1.069-6.662	0.036

## Discussion

In this study, we observed the persistence of HPV in 43 (43%) patients in total, and it was found to be more common in the lactobacilli depletion group. Although a decrease in the number of lactobacilli was observed in 21.1% of women in the group without HPV persistence, this rate was found to be significantly higher in the group with HPV persistence (46.5%, p=0.007). Our findings suggest that a decrease in lactobacilli in the vaginal microbiota is associated with persistent HPV infection. However, because of our modest sample size, further studies are needed.

HPV infection is the main cause of cervical cancer, which is the fourth most common cancer in women worldwide, while it ranks ninth in Turkey [10,11]. The most important point is that all cases of cancer are preventable with vaccination programs, early diagnosis, and effective management. In this study, the relationship between HPV persistence, which is the most important risk factor for cervical cancer, and cervicovaginal swab findings was compared. According to this comparison, a significant relationship was found between lactobacillus reduction in vaginal flora and HPV persistence. Besides, in multivariate analysis, this relationship was observed to continue regardless of age and menopause status.

Primary HPV screening is documented to be the most effective screening modality in cervical cancer screening [12,13]. However, a standardized triage and risk assessment of women after this screening modality has not been established yet. The risk of progression to cervical cancer is mediated through the persistence of HPV infection. Unfortunately, the risk factors for HPV persistence have not been clearly defined yet. Therefore, efforts to define actors associated with HPV persistence are of great clinical importance.

Although infection with the HPV may eventually result in cervical carcinogenesis, the most likely clinical possibility for the patient is to become negative over time without any disease. Many factors, especially immunity, can be held responsible for this process [14,15]. However, the risk factors affecting the progression of cervical carcinogenesis remain the subject of research. The hypothesis that chronic inflammation may promote carcinogenesis is supported by the increased levels of inflammatory cytokines found in patients with cervical cancer or its precursor premalignant stages [16]. It has been documented that the microbiome plays a protective role in infectious diseases and is economically important in the follow-up of diseases in public health [17]. Recently, mechanisms related to the stabilization of microbiota-mediated epithelial immunity have been described. The microbiota has been shown to have regulatory effects on immune system cells such as macrophages, T lymphocytes, and dendritic cells [18]. Moreover, the microbiota is quite effective in immune function by the development of many diseases in the case of dysbiosis, which means the breakdown of the microbiota [19]. However, there is a lack of evidence regarding the role of microbiota in the course of HPV infection. In this study, we documented that HPV persistence is more common in women with lactobacilli depletion. This result indicates that microbiota analysis in women with HPV infection may define the high-risk group for the persistence of HPV. Another hypothetical inference is the possible role of treatment with probiotic agents in cases with HPV persistence. To our knowledge, there have been no prospective studies for this aim.

Several specific types of lactobacillus were documented to dominate healthy vaginal microbiota in women [20]. Therefore, we hypothesized that the quantitative evaluation of the overall lactobacillus population may be a quick and easy way to define a subgroup of women who are at risk of HPV persistence after being recruited from primary HPV screening. Previously, Caselli et al. showed a high dysbiotic microbiome and vaginal proinflammatory cytokine increase in the group with CIN. However, they found a decrease in dysbiosis and inflammatory cytokines and a specific increase in Lactobacillus crispatus in particular in women whose HPV-related cervical lesion was surgically removed [21]. In our study, following this finding, we observed a decrease in the number of lactobacilli observed in 21.1% of women in the group without HPV persistence, and this rate was found to be significantly higher in the group with HPV persistence. However, in our study, there were no significant differences between the median values of the Nugent scores.

According to Kumari et al., the loss of the interrelationship between the cervicovaginal microbiota and the host leads to increased susceptibility to HPV infection. The progression to cervical neoplasia is a multistep process that is regulated by cellular and epigenetic changes. Exosomes derived from infected cells play an important role in pathological development and progression to cervical neoplasia, as they contain certain molecules that can facilitate cellular transformation [22]. In the study by Kwasniewski et al., cervical microbiota swabs taken from 250 women were evaluated, assuming that dysbiosis in the vaginal microbial environment is associated with cervical carcinogenesis caused by HPV. Cervical swabs from healthy volunteers were characterized by L. crispatus, Lactobacillus iners, and Lactobacillus taiwanensis, but not Gardnerella vaginalis and Lactobacillus acidophilus. In samples from patients with low-grade squamous intraepithelial lesions (LSIL), the predominant bacterial species were L. acidophilus and L. iners, but L. crispatus was not detected. In the samples taken from women with high-grade squamous intraepithelial lesion (HSIL), it was determined that G. vaginalis and L. acidophilus were abundant, but L. taiwanensis, L. iners, and L. crispatus were not found. The results showed that HPV-caused cervical cancer development is associated with a high diversity of the vaginal microbiota, which is involved in controlling viral persistence and, therefore, indicative of disease prognosis [23]. As a similar result, in a review including 1,230 cases, when the relationship of high-risk HPV-related lesions with lactobacilli was analyzed, it was concluded that L. crispatus was associated with decreased detection of high-risk HPV infection and L. crispatus had a critical effect against HPV infections. It has been underlined that it can be a protective factor [24].

Underlying vaginal DNA microbiological testing showed the presence or absence of bacterial vaginosis, other vaginal infections, or normal vaginal microbiota. Vaginal microbiota disorder was detected in 23 (71.9%) of the women with cervical cancer included in the study. Normal vaginal microbiota was detected in the remaining nine (28.1%) women. It has been demonstrated that bacterial dysbiosis, characterized by the predominance of G. vaginalis alone or in complex with other pathogens, and the deficiency of concomitant vaginal Lactobacillus species, may be an HPV-dependent cofactor for the development of cervical neoplasia [25].

As, in the current literature, there is no study evaluating the relationship between vaginal flora and HPV persistence in the follow-up of patients detected by the HPV screening program, our study is pioneering research conducted for this purpose. Another strength of our study was that all cervicovaginal swaps were collected before cervix evaluation and all of our cases were in routine follow-up with no extra excisional treatment at the time of initial evaluation. However, the limited number of patients in our study was the limitation of our study. It can be also a controversial topic whether an HPV infection was the cause of dysbiosis or dysbiosis was a risk factor for developing a persistent HPV infection. Another important research question is this: Does probiotic treatment have a place in the treatment of HPV persistence? 

## Conclusions

In conclusion, a decrease in lactobacilli in the cervicovaginal microbiota is associated with the persistence of HPV in these patients, regardless of age and menopausal status in this study. This finding is especially important for defining optimal management guidelines for women being recruited after an HPV screening program. Interventions for the vaginal microbiota may come to the fore in the management of HPV infection and cervical lesions caused by it. Therefore, further prospective studies are needed.
